# Skin Biopsies in Practice: Insights from a Pediatric Dermatopathology Study in a Greek Tertiary Center

**DOI:** 10.7759/cureus.103166

**Published:** 2026-02-07

**Authors:** Alexios Alexopoulos, Dimitrios Ntokos, Lamprini Nasi, Louiza Kontara, Pavlos Sarafis, Christina Stefanaki, Christina Kanaka-Gantenbein, Kalliopi Stefanaki

**Affiliations:** 1 First Department of Pediatrics, School of Medicine, National and Kapodistrian University of Athens, Agia Sophia Children's Hospital, Athens, GRC; 2 Department of Surveying and Geoinformatics Engineering, School of Engineering, University of West Attica, Athens, GRC; 3 Department of Pediatrics, North Middlesex University Hospital, London, GBR; 4 Department of Nursing, University of Thessaly, Lamia, GRC; 5 Department of Dermatology, School of Medicine, National and Kapodistrian University of Athens, Agia Sophia Children's Hospital, Athens, GRC; 6 Department of Pathology, School of Medicine, National and Kapodistrian University of Athens, Agia Sophia Children's Hospital, Athens, GRC

**Keywords:** biopsy indications, clinicopathologic correlation, diagnostic accuracy, histopathology, pediatric skin lesions, spitz tumor

## Abstract

Pediatric dermatologic conditions often present with overlapping clinical features, complicating accurate diagnosis and potentially delaying appropriate treatment. This retrospective study aimed to evaluate the diagnostic utility of pediatric skin biopsies by assessing clinicopathologic concordance and identifying cases in which histopathologic analysis led to diagnostic reclassification. Medical records of 140 children (≤14 years) who underwent skin biopsy at a tertiary-care center in Greece between February 2021 and November 2024 were retrospectively reviewed. Most biopsies (126/140, 90.0%) were performed for diagnostic or therapeutic purposes, while 14/140 (10.0%) were cosmetic. The most common histologic diagnoses were tumors and cysts (63/140, 45.0%), dermatitis (38/140, 27.1%), and nevi (14/140, 10.0%). Clinicopathologic concordance was observed in 125/140 cases (89.3%), whereas diagnostic reclassification occurred in 15/140 cases (10.7%), most frequently involving pityriasis lichenoides et varioliformis acuta, lupus profundus, infantile facial aseptic granuloma, and lymphomatoid papulosis. These findings underscore the diagnostic value of pediatric skin biopsies and support their earlier and more systematic integration into pediatric dermatologic care to enhance diagnostic accuracy and improve clinical outcomes.

## Introduction

In recent decades, pediatric dermatopathology has evolved from a diagnostic adjunct into a critical interpretive discipline, enabling clinicians to navigate the blurred margins between benign eruptions and serious neoplasms. Greek children, exposed to a combination of Mediterranean genetic diversity and region-specific environmental factors, often present with dermatologic phenotypes that resemble adult patterns while following distinct developmental trajectories [1-3].

Despite its diagnostic precision, skin biopsy remains selectively employed in pediatric practice. Procedural discomfort, limited patient cooperation, and caregiver apprehension frequently delay or prevent tissue sampling [4-6]. In parallel, general pediatricians who lack immediate access to dermatopathology consultation may rely on empiric treatment and clinical judgment, an approach that risks obscuring rare, atypical, or evolving pathologies [7-9]. This hesitation is reflected in the literature; globally, biopsy incidence in children rarely exceeds 2%, and in rural or resource-limited settings, it often falls well below 1%, as reported across multiple population-based studies [5,6].

Several retrospective analyses from Turkey, India, and Nigeria have highlighted substantial diagnostic variability, with reported clinicopathologic discordance rates ranging from 7% to 25%, alongside frequent high-stakes diagnostic reclassifications [10-12]. Studies from Central Europe and Switzerland further underscore the complexity of histologic interpretation in pediatric skin disease, particularly in distinguishing inflammatory mimics from atypical proliferations [13,14]. In response, recent calls for structured multidisciplinary review boards (MDTs) have gained traction, demonstrating improved clinicopathologic concordance and reduced medico-legal exposure [15].

Well-recognized diagnostic pitfalls include Spitz tumors misdiagnosed as melanoma, pilomatricomas mistaken for cystic lesions, and infantile facial aseptic granulomas (IFAGs) confused with inflammatory papules, all of which have been increasingly reported in pediatric dermatology series [16-18]. Similarly, vascular tumors such as pyogenic granulomas, frequently excised under clinical suspicion, require careful clinicopathologic correlation, particularly in ambiguous surgical contexts [19].

In Greece, however, the pediatric skin biopsy landscape remains largely undocumented. While individual case reports exist, no comprehensive dataset has systematically mapped the local incidence, diagnostic yield, or reclassification rate of pediatric cutaneous biopsies, leaving both epidemiologic insight and clinical guidance fragmented.

The present study seeks to address this gap by analyzing a three-year cohort of children biopsied at Greece’s largest tertiary pediatric hospital. The primary objective was to evaluate clinicopathologic concordance in pediatric skin biopsies, while secondary objectives included identifying patterns of diagnostic reclassification and biopsy indications in a tertiary care setting. It examines not only which lesions were sampled and for what indications, but also how frequently histopathologic evaluation altered the initial clinical impression and in what direction. By clarifying these patterns, the study aims to inform more timely biopsy referral, refine diagnostic pathways, and support evidence-based decision-making in pediatric dermatologic care, ultimately reducing the risk of delayed or inaccurate diagnosis.

## Materials and methods

Study design and setting

This retrospective observational study was conducted at the Pediatric Dermatology Unit of the Agia Sophia Children’s Hospital in Athens, Greece, a national tertiary care referral center for pediatric dermatologic conditions, between February 2021 and November 2024. The study was reviewed and approved by the Scientific Council of the Agia Sophia Children’s Hospital (protocol no. 58342/12.04.2025). All procedures were conducted in accordance with the principles of the Declaration of Helsinki. Written informed consent for procedures was obtained from the parents or legal guardians, and verbal or written assent was sought from children deemed developmentally able to participate in the decision-making process. All data were collected and analyzed in anonymized form.

Study population and clinical assessment

The medical records of all pediatric patients aged ≤14 years who underwent skin biopsy during the study period were systematically reviewed. Inclusion criteria encompassed biopsies performed for (i) diagnostic clarification, (ii) therapeutic management (e.g., complete lesion excision), or (iii) aesthetic concerns, particularly in the case of visible or pigmented lesions associated with parental anxiety. Biopsies performed outside the institution or lacking sufficient clinical or histopathologic documentation were excluded.

All patients underwent a comprehensive clinical assessment prior to biopsy, including evaluation of general health status, lesion morphology, anatomical distribution, symptomatology, and any associated systemic features. Dermoscopic images and clinical photographs were obtained when deemed clinically necessary. Each case was independently reviewed by two board-certified pediatric dermatologists, both of whom participated in biopsy decision-making and site selection. This dual-assessor approach minimized interobserver variability and enhanced diagnostic reliability, in line with previously published best practices [15].

Biopsy procedure and histopathologic evaluation

All biopsies were performed under local anesthesia using 3-mm or 4-mm punch instruments or excisional techniques, depending on lesion characteristics and anatomical location. Pediatric-specific considerations, including thinner skin, procedural anxiety, and anatomical constraints, were incorporated into instrument selection and anesthesia planning, in accordance with pediatric biopsy guidelines [20]. All specimens were formalin-fixed and evaluated by board-certified dermatopathologists with expertise in pediatric cutaneous pathology.

Each biopsy was categorized into one of six diagnostic groups: (i) dermatitis, (ii) tumors or cystic lesions, (iii) exanthematous diseases, (iv) autoimmune bullous diseases, (v) vasculitis, and (vi) nevi, including congenital, dysplastic, or sebaceous variants. Diagnostic concordance was assessed by comparing the pre-biopsy clinical impression with the final histopathologic diagnosis. Cases in which histopathology contradicted or substantially revised the initial clinical diagnosis were categorized as discordant or reclassified, in accordance with recent pediatric dermatopathology studies [12,14,21]. Diagnostic reclassification was defined as any histopathologic diagnosis that contradicted or substantially modified the initial clinical impression and had potential implications for patient management.

Statistical analysis

Descriptive statistics were used to summarize clinical and histologic data. Categorical variables were reported as absolute frequencies and percentages, while continuous variables were expressed as means, medians, standard deviations (SDs), and interquartile ranges (IQRs), as appropriate. Clinicopathologic concordance was expressed as the percentage of cases in which clinical and histologic diagnoses were aligned. All patient data were anonymized and handled in accordance with institutional data protection protocols.

## Results

Patient characteristics and biopsy indications

Over a 46-month period, 140 children (aged ≤14 years) underwent skin biopsy. Boys predominated (83/140, 59.3%), yielding a male-to-female ratio of 1.46:1. The mean age at intervention was 6.0 ± 4.8 years (median: 4.9 years; range: 2 months to 14 years). During the study period, the Pediatric Dermatology Unit recorded a total of 9,230 dermatology outpatient visits. Thus, the cumulative biopsy incidence was 1.5% (140 biopsies out of these 9,230 visits).

Of the medically driven biopsies (n=126, 90.0%), indications included: (i) complete excision with histologic verification (66/126, 52.4%), (ii) diagnostic confirmation (35/126, 27.8%), and (iii) precautionary sampling to exclude serious pathology (25/126, 19.8%). An additional 14 biopsies (14/140, 10.0%) were elective cosmetic excisions, primarily for conspicuous nevi or pyogenic granulomas requested by appearance-conscious adolescents.

Histopathologic spectrum

Tumors and cysts were the most common diagnoses (63/140, 45.0%), outnumbering inflammatory dermatoses (38/140, 27.1%) by nearly two to one (Table 1). Within the tumor subset, pilomatricoma (epithelioma of Malherbe) was the most frequent entity (18/63, 28.6%). Spitz tumors ranked second (15/63, 23.8%), while pyogenic granuloma and atypical xanthogranuloma accounted for 12/63 (19.0%) and 11/63 (17.5%), respectively.

**Table 1:** Distribution of disease categories and histopathological diagnoses (N=140)

Disease category	Total patients per category, n	Percentage of total biopsies (n/N)	Histopathological diagnosis	Cases, n	Percentage of total biopsies (n/N)
Dermatitis	38	27.1	Pityriasis lichenoides et varioliformis acuta (PLEVA)	14	10.0
			Psoriasis	12	8.6
			Lichen planus	3	2.1
			Morphea	6	4.3
			Lupus	2	1.4
			Netherton syndrome	1	0.7
Tumors / Cysts	63	45.0	Spitz tumor	15	10.7
			Pyogenic granuloma	12	8.6
			Epithelioma of Malherbe (pilomatricoma)	18	12.9
			Atypical xanthogranuloma	11	7.9
			Infantile facial aseptic granuloma (IFAG)	7	5.0
Exanthematous diseases	8	5.7	Viral exanthem	4	2.9
			Drug-related exanthematous eruption	2	1.4
			Lymphomatoid papulosis	1	0.7
			Anaplastic large T-cell lymphoma	1	0.7
Autoimmune bullous diseases	5	3.6	Various entities	5	3.6
Vasculitis	12	8.6	Predominantly IgA vasculitis	12	8.6
Nevi	14	10.0	Proliferative nodules in congenital melanocytic nevi	4	2.9
			Sebaceous nevi	3	2.1
			Epidermal nevi	2	1.4
			Dysplastic/atypical nevi	5	3.6

Among inflammatory dermatoses, pityriasis lichenoides et varioliformis acuta (PLEVA) was the leading diagnosis (14/38, 36.8%), followed by psoriasis (12/38, 31.6%) and morphea (6/38, 15.8%). Autoimmune bullous diseases were identified in 5/140 cases (3.6%), while classic exanthematous diseases accounted for 8/140 cases (5.7%). Vasculitis was observed in 12/140 cases (8.6%), predominantly IgA vasculitis. Nevi were identified in 14/140 cases (10.0%), including four proliferative nodules within congenital melanocytic nevi (4/140, 2.9%).

Clinicopathologic concordance and diagnostic reclassification

Clinicopathologic concordance was observed in 89.3% of cases (125/140), while histopathologic evaluation prompted diagnostic reclassification in 10.7% (15/140) (Table 2). Diagnostic reclassification occurred most frequently among tumors and cystic lesions (10/140, 7.1%), followed by inflammatory dermatoses (3/140, 2.1%) and exanthematous diseases (2/140, 1.4%).

**Table 2:** Diagnostic reclassification by disease category and histopathological diagnosis (N=140) PLEVA: pityriasis lichenoides et varioliformis acuta; IFAG: infantile facial aseptic granuloma

Disease category	Reclassified cases per category, n	Percentage of total biopsies (n/N)	Histopathological diagnosis	Reclassified cases, n	Percentage of total biopsies (n/N)
Dermatitis	3	2.1	Lupus profundus	1	0.7
			PLEVA	2	1.4
Tumors	10	7.1	Spitz tumor	5	3.6
			IFAG	2	1.4
			Atypical xanthogranuloma	3	2.1
Exanthematous diseases	2	1.4	Lymphomatoid papulosis	1	0.7
			T-cell anaplastic lymphoma	1	0.7

**Figure 1:**
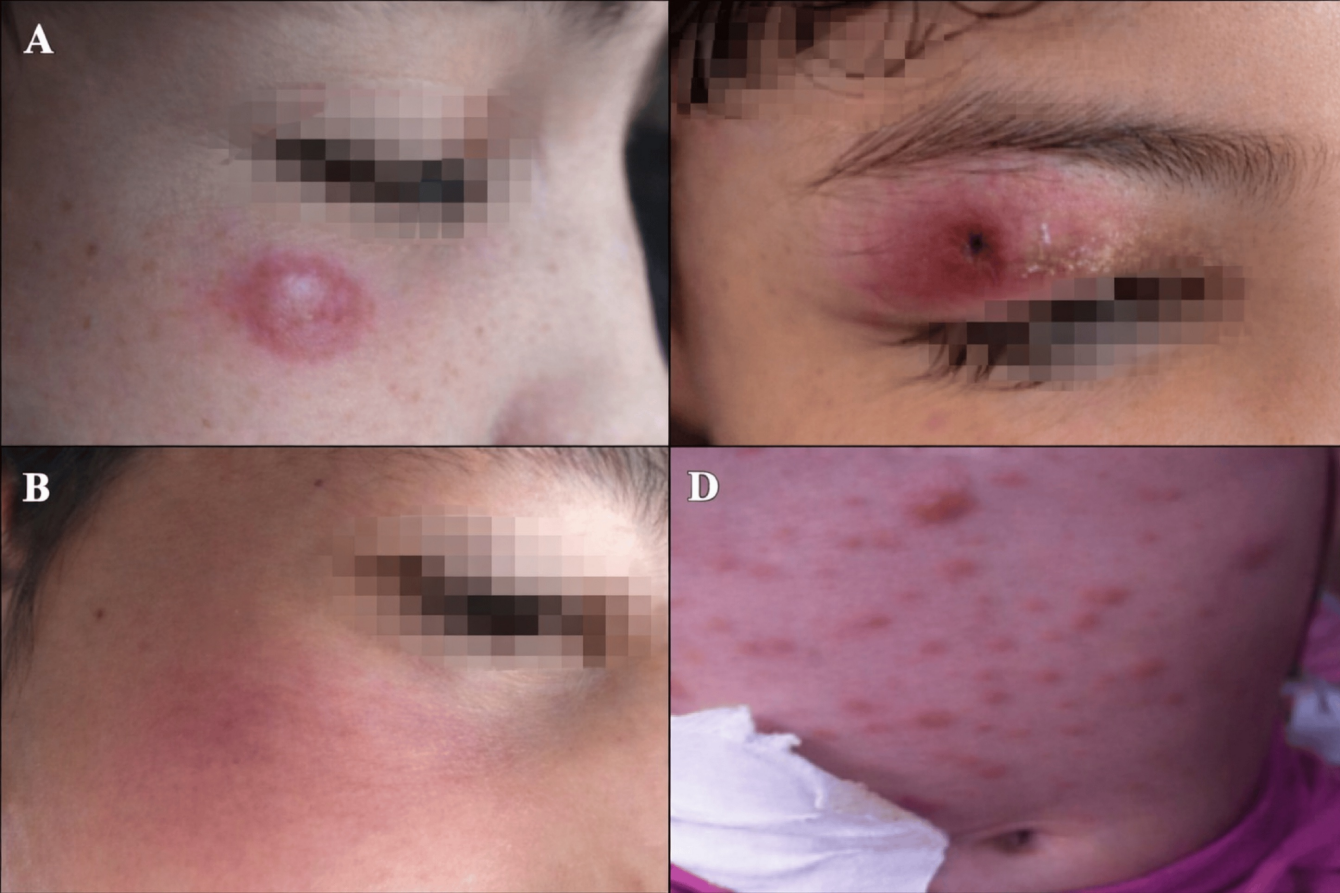
Representative pediatric skin lesions with clinicopathologic diagnostic reclassification (A) Infantile facial aseptic granuloma initially misdiagnosed as an insect bite reaction. (B) Lupus profundus initially misdiagnosed as panniculitis. (C) Lymphomatoid papulosis initially misdiagnosed as pityriasis lichenoides et varioliformis acuta (PLEVA). (D) Anaplastic large cell lymphoma initially interpreted as a postviral exanthem. Facial features were anonymized for patient confidentiality.

Among dermatitis cases, two lesions initially diagnosed as pityriasis rosea were reclassified as PLEVA, while one case of panniculitis was revised to lupus profundus. Within the tumor category, five lesions clinically suspected to represent melanoma were identified as benign Spitz tumors, two were reclassified as IFAL, and three as atypical xanthogranuloma. Two cases initially classified as exanthematous disease were ultimately diagnosed as lymphomatoid papulosis and anaplastic large T-cell lymphoma, respectively. Overall, diagnostic reclassification affected 15/140 cases (10.7%). Representative clinical examples of diagnostic reclassification are shown in Figure 1.

## Discussion

The cumulative biopsy incidence was 1.5% (140 biopsies), a figure comparable to reports from Northern Europe and the American Midwest [7,9] and higher than rates documented in rural India and Türkiye [6,8]. A biopsy incidence of 1.5% may appear modest; however, when compared with the 1-2% rates reported in Rochester, Zurich, and Ankara [7,13,14], the Greek figure lies comfortably within the global mid-range. The median age was 4.9 years. This early childhood skew, with occasional adolescent outliers, aligns with Swiss and Turkish cohorts reporting similar bimodal distributions [10,13,21]. 

More noteworthy is the diagnostic discordance rate observed in 15/140 children (10.7%), indicating that nearly one in 10 pediatric patients received a revised diagnosis following histopathologic evaluation. This follows the pragmatic “rule of eights,” whereby approximately 8-12% of pediatric skin lesions may defy initial clinical impressions, consistent with discordance rates reported in studies from Ankara, Zurich, and Lagos [12-14]. Comparable discordance rates have been reported in India (12.3%) and Nigeria (13.8%) [11,12], underscoring a consistent global reality in which pediatric skin diseases frequently defy initial clinical impressions and histopathology remains indispensable for diagnostic clarification. These findings challenge the assumption that most childhood rashes can be safely managed without histologic confirmation and reinforce the role of biopsy in suspected or persistent cases.

Viewed in aggregate, the core metrics of this cohort, including a mean age at biopsy of 6.0 years, a predominance of diagnostic or therapeutic indications in 126/140 cases (90.0%), and a histopathologic spectrum dominated by tumors and cystic lesions in 63/140 cases (45.0%), are broadly consistent with large-scale pediatric dermatology series from Europe and Asia [10,13]. At the same time, the diagnostic reclassification observed in 15/140 cases (10.7%) highlights the limitations of purely clinical assessment and underscores the clinical relevance of biopsy in ambiguous or treatment-resistant presentations.

Regional epidemiology shaped the histologic spectrum in instructive ways. Pilomatricoma accounted for 18/63 tumors (28.6%), exceeding pyogenic granuloma (12/63, 19.0%) in frequency, echoing the high prevalence reported in Indian and Turkish series [10,11]. This pattern potentially reflects Mediterranean UV exposure, keratin-pathway genetics, or referral bias. Systematic use of high-frequency ultrasound, marked by peripheral calcification and posterior acoustic shadowing, might have reduced unnecessary excisions [16,22-23].

The overrepresentation of pityriasis lichenoides et varioliformis acuta (PLEVA), observed in 14/38 dermatitis cases (36.8%), contrasts with South Asian cohorts, where lichen planus predominates [1,19]. Whether this reflects viral triggers, HLA variation, or increased diagnostic vigilance warrants further multicenter genomic research.

Several lesions initially suspected to be melanoma were ultimately diagnosed as benign Spitz tumors (5/15 reclassified neoplasms, 33.3%), mirroring findings by Calim-Gurbuz et al. [21]. Additional reclassifications included infantile facial aseptic granuloma initially thought to be arthropod reactions (2/15, 13.3%) and lupus profundus initially misdiagnosed as panniculitis (1/15, 6.7%). Two exanthematous presentations (2/15, 13.3%) were ultimately diagnosed as lymphomatoid papulosis and anaplastic large T-cell lymphoma, underscoring the potential severity of misclassification in pediatric dermatology. These diagnostic reversals further underscore the value of multidisciplinary team (MDT) reviews, as Tomasini et al. demonstrated their capacity to halve discordance rates and mitigate medico-legal risk [15]. Representative cases of reclassified lesions are illustrated in Figure 1.

Vascular tumors posed additional challenges. Cosmetic removals of pyogenic granuloma and capillary nodules accounted for a subset of tumor biopsies (12/63, 19.0%), echoing the broader pediatric vascular tumor spectrum described by Mathes and Frieden [24]. Case-based literature highlights the risk of overtreatment [24-26], and growing consensus now favors conservative management of many vascular proliferations, reserving excision for lesions with bleeding, functional compromise, or histologic uncertainty [26].

Why do pediatric biopsies remain underused? Procedural pain is only one barrier. Caregiver concerns, often amplified by misinformation, frequently override medical advice, while general pediatricians, lacking immediate access to dermatopathology, default to empiric therapy and observation [2,20]. In this context, the present findings support a two-tiered strategy: (i) integrating rapid-read dermatopathology services into tertiary centers, and (ii) disseminating visual triage tools (e.g., dermoscopy, high-frequency ultrasound, and red flag indicators) to peripheral clinics. Such interventions could foster earlier biopsy consideration and reduce diagnostic delays.

Education is equally critical. Cosmetic excisions of benign nevi accounted for 14/140 biopsies (10.0%) and often stemmed from parental anxiety rather than oncologic risk, a pattern observed in Swiss cohorts [13] and aligned with congenital nevus management guidelines by Alikhan et al. [3]. Structured counseling using evidence-based transformation risks may reduce unnecessary interventions while preserving clinical vigilance. From a clinical standpoint, these findings further reinforce the value of early biopsy consideration in pediatric patients with atypical, persistent, or treatment-refractory skin lesions.

Limitations and future directions

As a single-center retrospective study, our findings warrant cautious interpretation. Referral bias may have contributed to the overrepresentation of neoplastic lesions (63/140, 45.0%), while transient exanthems managed in primary care settings were likely underrepresented. Documentation limitations inherent to retrospective analysis precluded long-term follow-up of reclassified cases (15/140, 10.7%).

Future multicenter registries combining clinical, histopathologic, and genomic data could clarify whether the higher prevalence of pilomatricoma observed in Greek children (18/63 tumors, 28.6%)-relative to Asian populations where pyogenic granuloma predominates-is attributable to genetic predisposition, referral patterns, or diagnostic sensitivity. Emerging pediatric dermatopathology roadmaps increasingly advocate for this genomics-integrated approach to better understand regional patterns and improve diagnostic accuracy [27].

## Conclusions

This study highlights the diagnostic value of skin biopsy in pediatric dermatology, demonstrating that histopathologic evaluation can meaningfully alter initial clinical impressions. Diagnostic reclassification most frequently involved conditions initially considered benign or self-limiting, such as pityriasis rosea, arthropod reactions, and panniculitis, that were ultimately identified as PLEVA, IFAG, lupus profundus, or cutaneous lymphomas. These observations underscore the importance of maintaining a low threshold for biopsy in ambiguous or treatment-resistant presentations.

In this context, histopathology serves not only as a diagnostic endpoint but also as a safeguard against misclassification, particularly in settings with limited access to specialized pediatric dermatology expertise. The integration of non-invasive diagnostic tools and multidisciplinary collaboration may further support accurate diagnosis and appropriate management of pediatric dermatologic disease.

## References

[1] Sardana K, Mahajan S, Sarkar R (2009). The spectrum of skin disease among Indian children. Pediatr Dermatol.

[2] Paller AS, Mancini AJ (2022). Hurwitz Clinical Pediatric Dermatology: A Textbook of Skin Disorders of Childhood and Adolescence. 6th ed. Hurwitz Clinical Pediatric Dermatology: A Textbook of Skin Disorders of Childhood and Adolescence. 6th ed.

[3] Alikhan A, Ibrahimi OA, Eisen DB (2012). Congenital melanocytic nevi: where are we now? Part I. Clinical presentation, epidemiology, pathogenesis, histology, malignant transformation, and neurocutaneous melanosis. J Am Acad Dermatol.

[4] Afşar FŞ, Aktaş S, Diniz G (2011). The role of biopsy in pediatric dermatopathology. Turkderm Turk Arch Dermatol Venereol.

[5] Gimbel DC, Legesse TB (2013). Dermatopathology practice in Ethiopia. Arch Pathol Lab Med.

[6] Shetageri SN, Roopa AN, Parthiban R (2019). Histopathological spectrum of paediatric skin biopsies in a rural setup. Indian Journal of Pathology and Oncology.

[7] Storan ER, McEvoy MT, Wetter DA (2013). Pediatric hospital dermatology: experience with inpatient and consult services at the Mayo Clinic. Pediatr Dermatol.

[8] Şenel E, Yuyucu Karabulut Y, Karabulut HH (2014). Evaluation of skin biopsies in çankırı region: a two-year retrospective assessment. Turk J Dermatol.

[9] Landolt B, Staubli G, Lips U, Weibel L (2013). Skin disorders encountered in a Swiss pediatric emergency department. Swiss Med Wkly.

[10] Ozkanli S, Zemheri E, Zindanci I, Kuru B, Zenginkinet T, Karadag AS (2015). Three years of retrospective evaluation of skin biopsy results in childhood. North Clin Istanb.

[11] Vishwanath T, Kharkar V, Gole P, Mahajan S, Chikhalkar S (2023). A six-year retrospective analysis of skin biopsies in the pediatric and adolescent population performed at a tertiary health care center in India. Indian Dermatol Online J.

[12] Ndukwe CO, Eziagu UB, Eni AO (2024). Histopathologic spectrum and clinicopathologic concordance of pediatric skin biopsies: 18-year experience in a tertiary hospital in southeast Nigeria. Med J Dr DY Patil Vidyapeeth.

[13] Theiler M, Neuhaus K, Kerl K, Weibel L (2017). The spectrum of skin biopsies and excisions in a pediatric skin center. Eur J Pediatr.

[14] Çölgeçen E, Şahin S, Gürel G (2020). Clinicopathological correlation of skin biopsies in pediatric patients. J Ankara Univ Fac Med.

[15] Tomasini CF, Michelerio A, Isoletta E, Barruscotti S, Wade B, Muzzi A (2023). A clinico-pathological multidisciplinary team increases the efficacy of skin biopsy and reduces clinical risk in dermatology. Dermatopathology (Basel).

[16] Sun J, Fu LB, Xu JS, Han XF, Wei L (2023). Confused subcutaneous nodules in children: differential diagnosis of pilomatricoma in children. J Cosmet Dermatol.

[17] Zitelli KB, Sheil AT, Fleck R, Schwentker A, Lucky AW (2015). Idiopathic facial aseptic granuloma: review of an evolving clinical entity. Pediatr Dermatol.

[18] Weir SA, Amin S, Higgins A, Kelly D, Theos A (2023). Idiopathic facial aseptic granuloma: case series and review of histological findings. Proc (Bayl Univ Med Cent).

[19] Balasubramaniam P, Ogboli M, Moss C (2008). Lichen planus in children: review of 26 cases. Clin Exp Dermatol.

[20] Nandakumar G (2012). Skin biopsy in pediatric age group: special considerations. Ind J Paediatr Dermatol.

[21] Calim-Gurbuz B, Pehlivanoglu B, Soylemez-Akkurt T (2023). Skin lesions in children: evaluation of clinicopathological findings. Turk Patoloji Derg.

[22] Hwang JY, Lee SW, Lee SM (2005). The common ultrasonographic features of pilomatricoma. J Ultrasound Med.

[23] Li L, Xu J, Wang S, Yang J (2021). Ultra-high-frequency ultrasound in the evaluation of paediatric pilomatricoma based on the histopathologic classification. Front Med (Lausanne).

[24] Mathes EF, Frieden IJ (2026). Chapter 126. Vascular tumors. Fitzpatrick's Dermatology in General Medicine, 8e.

[25] Shirani AM, Tadayonnezhad P, Arzani S, Kiansadr SO, Kaviani N (2024). Laser excisional biopsy of bleeding tumor near newly erupted tooth in an 11-month-old patient under general anesthesia. Case Rep Dent.

[26] Bin Dlaim MS, Alhussein GA, Alqahtani RS, Almanea LT (2023). Conservative management of giant pyogenic granuloma post strabismus surgery: a case report and literature review. Cureus.

[27] Fraitag S (2024). New Insights in Paediatric Dermatopathology-2nd Edition (Editorial). Dermatopathology (Basel).

